# Polymyositis: The Comet Tail After COVID-19

**DOI:** 10.7759/cureus.26453

**Published:** 2022-06-30

**Authors:** Said Amin, Fawad Rahim, Mohammad Noor, Ayesha Bangash, Fazal Ghani

**Affiliations:** 1 Internal Medicine, Khyber Girls Medical College, Peshawar, PAK; 2 Internal Medicine, Hayatabad Medical Complex Peshawar, Peshawar, PAK

**Keywords:** coronavirus disease 2019 (covid-19), sars-cov-2 (severe acute respiratory syndrome coronavirus-2), muscle weakness, rheumatic disorder, polymyositis

## Abstract

Infectious agents have been implicated in the pathogenesis of autoimmune disorders for decades. Severe acute respiratory syndrome coronavirus 2 (SARS-CoV-2) is no exception. This became evident as the pandemic evolved. Once considered a respiratory pathogen only, SARS-CoV-2 is now linked to a variety of autoimmune rheumatic disorders such as rheumatoid arthritis, systemic lupus erythematosus, reactive arthritis, spondyloarthropathies, vasculitis, and inflammatory myopathy. Although the exact cause for muscle injury in the setting of coronavirus disease 2019 (COVID-19) is not established, autoimmune inflammatory damage is the most accepted mechanism. Moreover, SARS-CoV-2 can cause direct muscle damage and indirectly through a cytokine storm. Inflammatory polymyositis in relation to COVID-19 has seldom been reported in developing countries.

Here, we report a unique case of inflammatory polymyositis in a 52-year-old lady. The patient presented with muscle weakness, generalized body aches, and fatigue occurring four months after recovering from mild COVID-19. She had muscle weakness of Medical Research Council (MRC) grade 3/5 involving the shoulders and pelvic girdle with elevated muscle enzymes. Electromyography revealed an active irritable myopathic process consistent with inflammatory polymyositis. She underwent magnetic resonance imaging-guided muscle biopsy from the right thigh which revealed findings consistent with inflammatory myopathy. She was offered prednisolone and azathioprine. After four weeks of treatment, she had a remarkable improvement in her muscle strength to MRC grade 5/5.

## Introduction

Viruses have been linked to the initiation and modulation of immune responses in various autoimmune rheumatic illnesses. However, the processes by which viruses and autoimmune disorders interact remain unknown [1]. Coronavirus disease 2019 (COVID-19), which was once assumed to be a pulmonary ailment, is now considered a multisystemic disease that manifests differently in different patients and has a variable outcome [2].

The effects of severe acute respiratory syndrome coronavirus 2 (SARS-CoV-2) on the immune system and cytokine profiles are unclear. Several reports have been published describing the immunological alterations seen with COVID-19. These range from a maladaptive immune response and abnormal cytokine production to hyperactivation of T cells and increased numbers of activated monocytes and macrophages [3].

Antinuclear antibodies (ANA), anti-Sjogren syndrome-related antigen A antibody (anti-SSA), rheumatoid factor (RAF), lupus anticoagulant, and antibodies to interferon are found in 35.6%, 25%, 19%, 11%, and 11% of cases of COVID-19, respectively [4].

The leading causes of muscle involvement in COVID-19 include idiopathic inflammatory myositis, dermatomyositis, necrotizing autoimmune myositis, anti-synthetase syndrome-overlap myositis, and inclusion body myositis [5]. The most commonly reported mechanism of muscle weakness in COVID-19 is autoimmune inflammatory damage. Additionally, direct invasion of the muscles by SARS-CoV-2 and muscle damage due to cytokine storm have also been proposed [6]. Muscle involvement manifests as fatigue, exercise intolerance, myalgias, difficulty standing and walking, and progressive shortness of breath [7].

So far, nine cases of inflammatory polymyositis/dermatomyositis following COVID-19 have been reported. Of the nine cases, four have been reported from Europe and two each from the United States and Asia. Out of nine cases, five were female with a mean age of 55.6 years; most (seven cases) of them recovered [8]. Moreover, few cases of inflammatory polymyositis have been reported following COVID-19 vaccination [9]. Here, we report the case of a 52-year-old female who presented with inflammatory polymyositis four months after recovering from mild COVID-19.

## Case presentation

A 52-year-old hypertensive lady presented with complaints of shortness of breath, muscle weakness, generalized body aches, and fatigue for the last four months. The shortness of breath was gradual in onset but recently worsened to the extent that she could hardly perform her daily activities. There was no history of orthopnea, paroxysmal nocturnal dyspnea, and chest pain. The muscle weakness had started gradually four months ago and was predominantly in the proximal muscles. It was accompanied by body aches and fatigue. There was no diurnal variation in the muscle weakness. She had difficulty placing heavy objects on the shelf, combing her hair, rising from a sitting position, and walking up and down the stairs. There was no history of joint pain, swelling, stiffness, Raynaud’s phenomena, dysphagia, and skin rashes.

She had a reverse transcriptase-polymerase chain reaction-confirmed COVID-19 infection four months ago. The severity of COVID-19 was mild as per the World Health Organization (WHO) criteria. She received appropriate treatment and had an uneventful recovery.

She had no significant relevant medical or surgical history, and she was not taking any regular medications except paracetamol. Her travel history was not significant. She was not vaccinated against COVID-19.

On examination, her pulse was 80 beats per minute with a blood pressure of 120/70 mmHg, temperature of 98°F, respiratory rate of 14 breaths per minute, and oxygen saturation of 97% on room air. She had weakness involving proximal muscle groups of upper and lower limbs bilaterally, Medical Research Council (MRC) grade 3/5. There was no rash on the hands or face. The rest of her examination was unremarkable. She was admitted to the medical unit of Hayatabad Medical Complex Peshawar, Pakistan for further workup. Her initial investigations are summarized in Table 1.

**Table 1:** Investigations at the tertiary care hospital. ELISA: enzyme-linked immunosorbent assay; HBsAg: hepatitis B surface antigen; HCV: hepatitis C virus; HIV: human immunodeficiency virus; SARS-CoV-2: severe acute respiratory syndrome coronavirus 2; PCR: polymerase chain reaction; RBS: random blood sugar; TSH: thyroid-stimulating hormone

Investigation	Result	Reference range
WBC (×10^3^/µL)	13.0	4–11
RBC (×10^6^/µL)	4.66	4–6
Hb (g/dL)	11.8	11.5–17.5
HCT (%)	36.5	36–54
MCV (fL)	78.3	76–96
MCH (pg)	25.3	27–33
MCHC (g/dL)	32.3	33–35
Platelet count (×10^3^/µL)	553	150–450
Neutrophils (%)	65	7.2–11
Lymphocytes (%)	30	20–45
Monocytes (%)	02	2–10
Eosinophils (%)	02	0–6
CRP (mg/dL)	40.5	<0.5
Total bilirubin (mg/dL)	0.8	0.1–1.2
ALP (U/L)	244	<275
ALT (U/L)	132	10–41
CPK (U/L)	2,225	25–200
Urea (mg/dL)	29	10–40
Creatinine (mg/dL)	1.1	0.2–1.2
HBsAg (ELISA)	Non-reactive	Non-reactive
Anti-HCV (ELISA)	Non-reactive	Non-reactive
Anti-HIV (ELISA)	Non-reactive	Non-reactive
SARS-CoV-2 PCR	Negative	Negative
RBS (mg/dL)	110	60–150
TSH (mIU/L)	1.3	0.5–5.0
Na (mEq/L)	130	135–145
K (mEq/L)	5.2	3.5–5.5
Cl (mEq/L)	97.4	95–110
Urinalysis	Normal	
Chest X-ray	Normal	
Ultrasound of the abdomen and pelvis	Fatty liver and atrophic left kidney	

**Figure 1:**
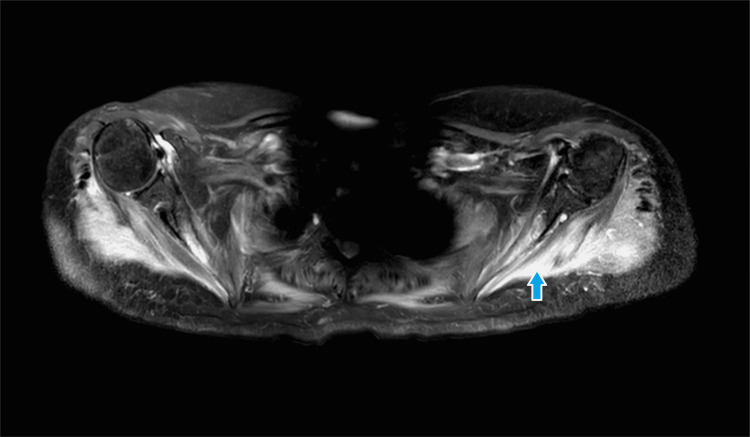
Magnetic resonance imaging (T2-weighted image) of shoulder joints showing high signals in shoulder girdle muscles.

**Figure 2:**
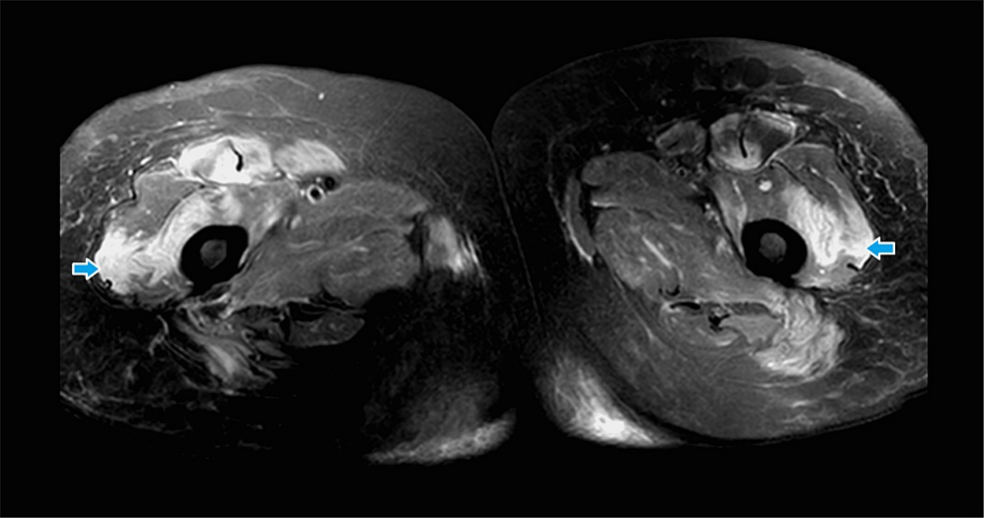
Magnetic resonance imaging (T2-weighted, axial view) of the thighs showing high signals in the muscles.

**Figure 3:**
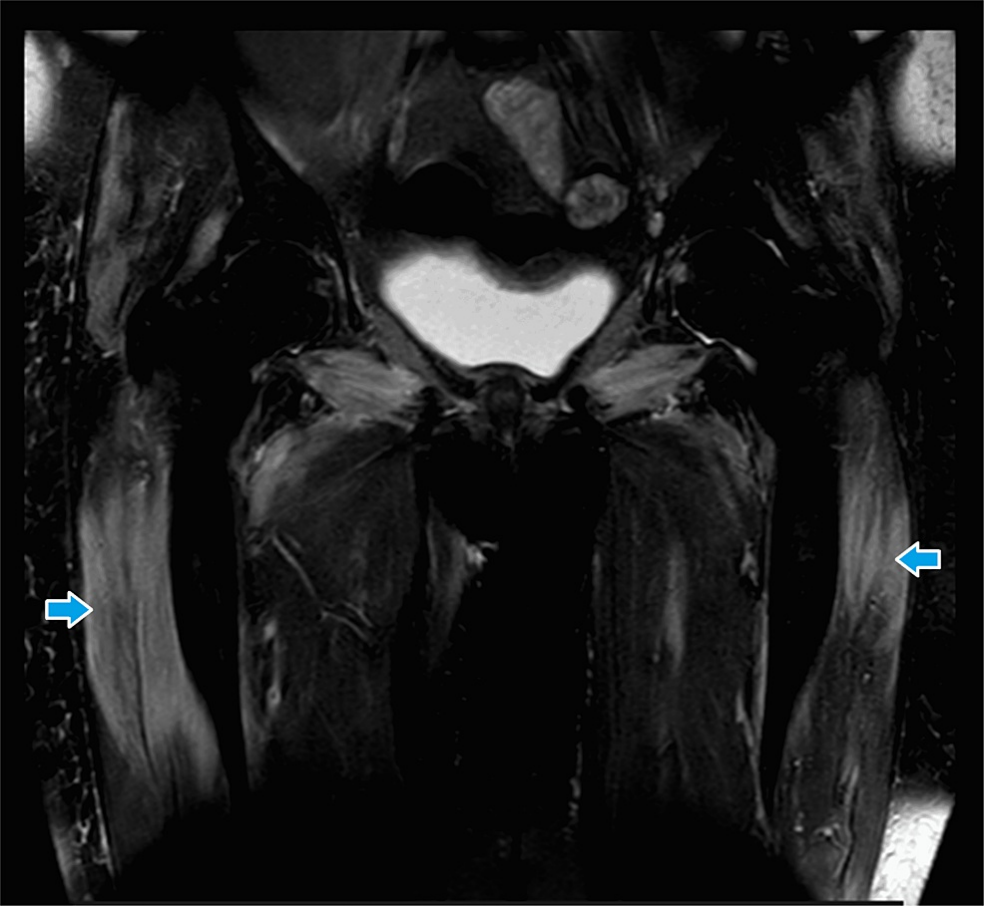
Magnetic resonance imaging (T2-weighted, coronal view) of the thighs showing high signals in muscles.

Her autoimmune profile including ANA, RAF, and anti-cyclic citrullinated peptides was negative. A further autoimmune screen could not be performed as the facilities were not available locally. She underwent a nerve conduction study and electromyography which showed an active irritable myopathic process consistent with inflammatory polymyositis. She was started on tablet prednisolone 60 mg per day along with calcium and vitamin D supplements. Azathioprine 50 mg twice a day was added to her regimen as a steroid-sparing agent. She underwent contrast-enhanced computed tomography of the chest, abdomen, and pelvis which showed enlarged fatty liver and atrophic left kidney. Given the electromyography findings, she underwent magnetic resonance imaging of the shoulder and hip muscles which revealed inflammatory changes in the muscles of the shoulder and pelvic girdle, chest, and anteromedial and lateral compartments of the thighs (Figures 1-3).

**Figure 4:**
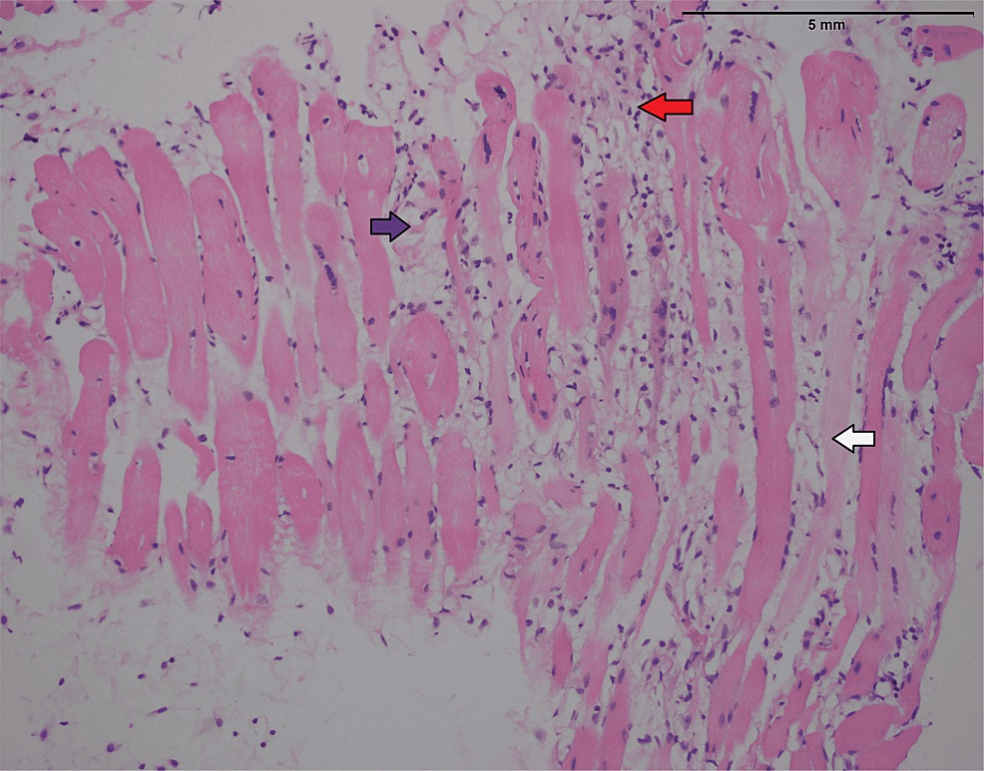
Hematoxylin and eosin stain (low-power view) showing skeletal muscle atrophy (white arrow), chronic inflammation (red arrow), and replacement by fibro-adipose tissue (blue arrow).

**Figure 5:**
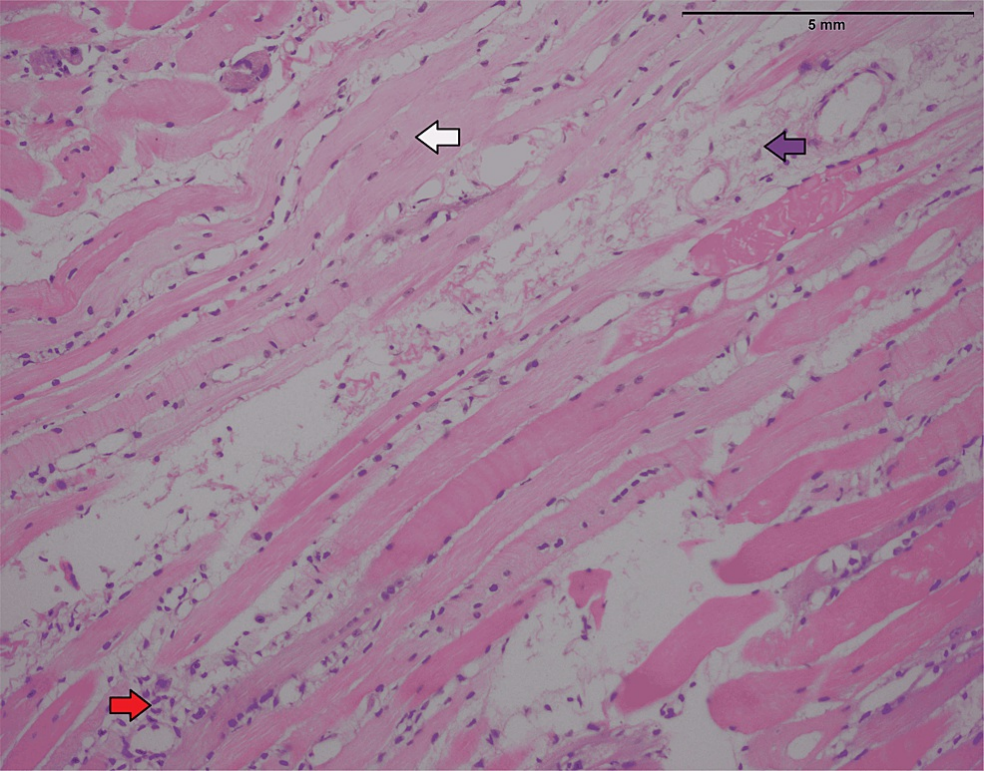
Hematoxylin and eosin stain (high-power view) showing skeletal muscles atrophy (white arrow) and replacement by fibro-adipose tissue (blue arrow) and chronic inflammation (red arrow).

She underwent a muscle biopsy from the right thigh. The histopathology report revealed lymphocytic infiltrate in the muscle, atrophy of the muscle fibers with the splitting of the fibers, vacuolization of cytoplasm, and internalization of nuclei, suggestive of inflammatory myopathy. The inflammatory cells were positive for cluster of differentiation 3 (CD3) and replacement of muscle fibers by fibro-adipose tissue (Figures 4, 5).

The patient was assessed daily during her in-patient stay for muscle power and vital capacity. On day 10, the power in her upper and lower limbs improved to the extent that she could walk without support. The changes in her laboratory parameters throughout her stay are summarized in Table 2.

**Table 2:** Laboratory Investigations during the hospital stay. CPK: creatinine phosphokinase; CRP: C-reactive protein

Investigation	Reference range	Day one	Day three	Day seven	Day ten
CPK (U/L)	25–200	2,225	1,498	840	632
CRP (mg/dL)	<0.5	40.5	32.4	21.7	12.2
Urea (mg/dL)	10–40	29	34	31	32
Creatinine (mg/dL)	0.2–1.2	1.1	1.1	1.0	0.8

The patient was discharged and advised to follow up in the medical outpatient clinic to assess her respiratory and motor system and titrate the steroid accordingly. After four weeks, she was reviewed in the outpatient clinic. She had a remarkable improvement in muscle strength of 5/5 on the MRC scale. Her creatinine phosphokinase (CPK) was 115 U/L. She was booked for a dual-energy X-ray absorptiometry scan as a baseline. The prednisolone was tapered to 50 mg daily aiming to reduce it to a 20 mg maintenance dose within four months.

## Discussion

As the COVID-19 pandemic unfolds, SARS-CoV-2 is behaving like a chameleon. Once thought to be a respiratory pathogen, it is now associated with a variety of systemic manifestations. COVID-19 is associated with extrapulmonary involvement, including autoimmune rheumatic disorders, for which an exact immunological mechanism needs to be determined. The initial assumption that COVID-19 is a stand-alone pulmonary infectious disease has significantly changed [10].

Only one case of COVID-19-related inflammatory polymyositis was documented in the literature by July 2020. Since then, multiple case reports and series have been published describing virus-induced inflammatory polymyositis linked to COVID-19. The involvement of muscles might range from asymptomatic muscle enzyme elevation to severe rhabdomyolysis [11]. It was reported in the initial cohort that 19% of patients with COVID-19 had inflammatory polymyositis, as evident by raised CPK [12]. Several cases of inflammatory polymyositis and rhabdomyolysis have been reported in association with SARS-CoV-2 infection and COVID-19 vaccination [1].

A systematic review by Gracia-Ramos et al. from December 2019 to September 2021 identified 99 patients who fulfilled the specific diagnostic criteria of rheumatic autoimmune disease. In total, 17 patients had inflammatory myopathies, and nine fulfilled the Peter and Bohan criteria. The mean age of the patients was 55.6 years (ranging from 38 to 77 years), and two-thirds of the cases were female. The time interval between COVID-19 and the development of inflammatory polymyositis varied from ten days to three months. The majority of cases (55.5%) had critical and severe COVID-19 [8].

A review by Saud et al. in 2021 found 23 cases of inflammatory polymyositis, with most cases reported in males, in contrast to Gracia-Ramos et al. The age ranged from 33 to 87 years [5]. A mini-review by Qian and Xu found seven cases of inflammatory polymyositis during 2021-2022, and five of them died due to critical COVID-19 [13].

In our case, a female aged 50 years developed inflammatory myositis four months after mild COVID-19. Even though idiopathic inflammatory polymyositis is more common among females, there is inconsistent evidence for the gender predilection of inflammatory polymyositis following COVID-19 [14]. The clinical characteristics of this case are consistent with the largest case series by Gracia-Ramos et al. and in contrast with the case series by Saud et al. [5,8]. While most cases of inflammatory polymyositis have followed severe and critical COVID-19, this case is unique because it developed four months after an uneventful recovery from mild COVID-19 illness. We acknowledge the limitation of this case report to draw firm conclusions that the inflammatory polymyositis was due to COVID-19 four months ago or it was merely a chance association between the two. Further research in the form of case-control studies and cohort studies would resolve the issue. This case will add to the reported cases of inflammatory myopathies following COVID-19, which are seldom reported from developing countries like Pakistan.

## Conclusions

COVID-19 is no longer an acute illness limited to the respiratory system. It may involve other systems at the time of the initial illness, several months after recovery from the acute infection, and even after vaccination for COVID-19. It can lead to fatality even if the person survives the acute infection. Physicians should be vigilant to look for multisystemic involvement during COVID-19 illness. Moreover, all COVID-19 patients should be educated and followed for a long time after recovery lest they develop late multisystemic manifestations.
